# Enhancing Sesquiterpenoid Production from Methane via Synergy of the Methylerythritol Phosphate Pathway and a Short-Cut Route to 1-Deoxy-D-xylulose 5-Phosphate in Methanotrophic Bacteria

**DOI:** 10.3390/microorganisms9061236

**Published:** 2021-06-07

**Authors:** Anh Duc Nguyen, Diep Ngoc Pham, Tin Hoang Trung Chau, Eun Yeol Lee

**Affiliations:** Department of Chemical Engineering (Integrated Engineering), Kyung Hee University, Yongin-si 17104, Gyeonggi-do, Korea; nguyenducanh01051991@gmail.com (A.D.N.); pndiep.93@gmail.com (D.N.P.); chttin93@gmail.com (T.H.T.C.)

**Keywords:** sesquiterpenoids, *Methylotuvimicrobium alcaliphilum* 20Z, methylerythritol phosphate pathway, α-bisabolene, methane

## Abstract

Sesquiterpenoids are one of the most diverse classes of isoprenoids which exhibit numerous potentials in industrial biotechnology. The methanotrophs-based methane bioconversion is a promising approach for sustainable production of chemicals and fuels from methane. With intrinsic high carbon flux though the ribulose monophosphate cycle in *Methylotuvimicrobium alcaliphilum* 20Z, we demonstrated here that employing a short-cut route from ribulose 5-phosphate to 1-deoxy-d-xylulose 5-phosphate (DXP) could enable a more efficient isoprenoid production via the methylerythritol 4-phosphate (MEP) pathway, using α-humulene as a model compound. An additional 2.8-fold increase in α-humulene production yield was achieved by the fusion of the nDXP enzyme and DXP reductase. Additionally, we utilized these engineering strategies for the production of another sesquiterpenoid, α-bisabolene. The synergy of the nDXP and MEP pathways improved the α-bisabolene titer up to 12.24 ± 0.43 mg/gDCW, twofold greater than that of the initial strain. This study expanded the suite of sesquiterpenoids that can be produced from methane and demonstrated the synergistic uses of the nDXP and MEP pathways for improving sesquiterpenoid production in methanotrophic bacteria.

## 1. Introduction

Isoprenoids are the largest family in natural products which are found as secondary metabolites in many organisms including plants, animals, and microbes and exhibit a wide range of applications in pharmaceuticals, nutraceuticals, flavors, cosmetics, food additives, and biofuels [1]. In the isoprenoid family, sesquiterpenoids are the largest and the most diverse subgroup with important medical and industrial properties [2]. However, there are several challenges for isoprenoid production via chemical synthesis or plant extraction [3]. While extraction from plants has several disadvantages such as low productivity, weather dependency, and waste production, chemical synthesis requires many steps with expensive procedures and environmental hazardous catalysts. Thus, the development of microbial cell factories for the production of diverse isoprenoids has been drawing a lot of attention in biotechnology for decades due to these challenges [2,3,4].

The microbial production described so far has used sugar as the major feedstock. However, due to increased sugar prices, inexpensive and non-food carbon feedstocks, therefore, are needed [5,6,7]. Methane is considered as an alternative carbon feedstock for industrial biomanufacturing because of its relatively high abundance and cheap price [7,8]. Additionally, methane has a degree of reduction per carbon of 8 which is much higher than that of 4 in glucose, indicating that methane has more electrons per carbon atom and those electrons can be used to enhance product yields [8]. Methanotrophs-based methane bioconversion does not compete with food production, which contrasts with other microbial hosts employing biomass-derived substrates, enabling its potential in industrial manufacturing for the sustainable bioproduction of isoprenoids as well as of other biochemicals [9,10]. *Methylotuvimicrobium alcaliphilum* 20Z is a model methanotrophic system because of its promising biotechnological potential with the availability of genetic tools, omics and physiological datasets, making it a potential methanotrophic biocatalyst for methane conversion [11,12,13]. However, the experience of generating isoprenoids from methane in methanotrophic bacteria as well as of this particular biocatalyst is very limited [14,15]. Some methanotrophic strains naturally produce carotenoids, an important class of isoprenoids, such as *Methylomonas* sp. DH-1 and *Methylomonas* sp. ZR1 [16,17]. In contrast, some are unable to synthesize isoprenoids without metabolic engineering, although they have full enzymes in the MEP pathway [14]. Previously, *Methylomonas* sp. 16a was demonstrated to produce a small amount of astaxanthin from methane by expressing an astaxanthin-synthesizing gene cluster [18]. Furthermore, *M. alcaliphilum* 20Z, a halophilic methanotroph, was engineered for α-humulene production via the methylerythritol 4-phosphate (MEP) pathway [19]. This demonstrates an important step forward in methanotrophic biocatalysts for the conversion of methane to isoprenoids. However, low titers of sesquiterpenoids highlight the need for more advanced engineering strategies to improve the production of isoprenoids using this promising biocatalyst.

DXP, 1-deoxy-D-xylulose 5-phosphate, has a similar structure to pentose phosphates. However, DXP is naturally produced by condensation of pyruvate (PYR) and glyceraldehyde 3-phosphate (G3P) using DXP synthase (Dxs) with the loss of one CO_2_ [16]. Dxs is a well-known gatekeeper for the MEP pathway which is regulated at both the transcriptional and the translational levels and the feedback is inhibited by prenyl phosphates [20]. To improve the carbon flux entering the MEP pathway, a short cut from C5 sugars to DXP (nDXP pathway) is a potential strategy since it possesses several advantages such as enhancement of the carbon conservation yield and avoidance of regulatory mechanisms [21]. With a high carbon flux through the ribulose monophosphate (RuMP) cycle in *M. alcaliphilum* 20Z for C5 regeneration, we demonstrated here the potential use of the nDXP pathway for improving the carbon flux through the MEP pathway, subsequently enhancing sesquiterpenoid production from methane in *M. alcaliphilum* 20Z. This study also represents the first demonstration of microbial production of α-bisabolene from methane.

## 2. Materials and Methods

### 2.1. Strains and Plasmids

The list of all strains used in this study is provided in Supplementary Table S1. *Methylotuvimicrobium* (formerly known as *Methylomicrobium*) *alcaliphilum* 20Z and the engineered strains were cultivated in 500-mL baffled flasks sealed with screw caps containing 50 mL nitrate mineral salt (NMS) medium with compositions described in detail by Ojala et al. at 30 °C and 230 rpm using methane as a carbon source [22]. Methane was supplied to a final concentration of 50% (*v*/*v*) by gas substitution using a gas-tight syringe, and the headspace was refreshed daily. Kanamycin with the final concentration of 50 µg/mL was used for selecting recombinant methanotrophs and *Escherichia coli* with recombinant plasmids.

The primers and procedures used for plasmids construction are provided in Supplementary Table S2, with the construction of platform plasmid pHM03 thoroughly described by Nguyen et al. [19]. The different recombinant plasmids were constructed with distinct genes but the constitutive promoter, P_tac_, was kept consistently for driving the transcription. These recombinant plasmids were transformed into the host strain using electroporation described in detail by Nguyen et al. [11]. In brief, cells were harvested by centrifugation and washed twice using cold water. An amount of 100 µL of distilled sterile water was used to resuspend the cell pellets. Then, 50 µL cell suspension was mixed with the DNA plasmid and put into a cold 1-mm gap cuvette for electroporation at parameters of 1.3 kV, 25 µF, and 200 Ω. The electroporated cells were then recovered in 10 mL NMS overnight and spread on selective plates. The original *ribB* sequence from *Methanococcus jannaschii* was codon-optimized to maximize the codon adaptation index between the codon usage of *ribB* and the codon usage of all ORFs from *M. alcaliphilum* 20Z as described previously [19].

### 2.2. Production of α-Humulene and α-Bisabolene and the Analytical Method

Two-phase flask cultivation consisting of the NMS medium as the aqueous phase to cultivate methanotrophs and 20% (*v*/*v*) dodecane as the organic overlay to extract α-humulene and α-bisabolene produced from the engineered strains in situ was performed in all of the experiments as described in our previous report [19]. Briefly, precultures were grown in 10 mL NMS medium and then inoculated into 500-mL baffled flasks containing 40 mL fresh media to achieve OD_600_ of 0.1 and 10 mL (20% *v*/*v*) dodecane. Sampling was conducted after 96-h cultivation by centrifugation of 50 mL liquid culture at 3220 g for 10 min, and then the upper dodecane layer was collected for α-humulene and α-bisabolene quantification. An Agilent 5977B 5977E GC/MS system (Santa Clara, CA, USA) was used to analyze and quantify α-humulene and α-bisabolene according to previous protocols [23,24]. For quantification of α-humulene and α-bisabolene, standard curves were made from analytical standards dissolved in dodecane which was purchased from Sigma-Aldrich (St. Louis, MO, USA) and Alfa Aesar (Ward Hill, MA, USA). Due to the presence of other bisabolene isomers, the contribution of α-bisabolene to the molarity of 27.52 ± 0.27% in the commercial standard was calculated previously [24].

### 2.3. Calculation of the Maximum Theoretical Molar Yield Using a Genome-Scale Model

The *i*IA407 genome-scale model (GSM) of *M. alcaliphilum* 20Z was used for in silico calculation [12]. The *i*IA407-nDXP model was constructed by adding heterologous reactions for the nDXP reaction and an exchange reaction for converting isopentenyl pyrophosphate (IPP) into *i*IA407 by Cobrapy (Appendix A) [25]. The maximum theoretical molar yield of IPP (MTMY_IPP_) was calculated by means of the flux balance analysis using OptFlux [26].

## 3. Results and Discussion

### 3.1. Enhancing α-Humulene Production via the Synergy of the MEP and nDXP Pathways

**Figure 1:**
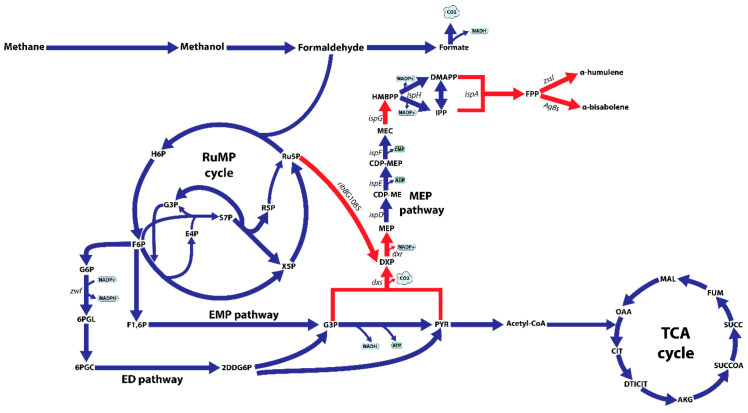
Schematic overview of the central metabolism of *M. alcaliphilum* 20Z on methane, including the ribulose monophosphate (RuMP) cycle, the Embden–Meyerhof–Parnas (EMP) pathway, the Entner–Doudoroff (ED) pathway, the TCA cycle, endogenous isoprenoid synthesis via the methylerythritol 4-phosphate pathway (MEP). Abbreviations: H6P, hexulose 6-phosphate; F6P, fructose 6-phosphate; X5P, xylulose 5-phosphate; Ru5P, ribulose 5-phosphate; E4P, erythrose 4-phosphate; S7P, sedoheptulose 7-phosphate; R5P, ribose 5-phosphate; F1,6P, fructose 1,6-bisphosphate; G6P, glucose 6-phosphate; 6PGL, 6-phosphogluconolactonase; 6PGC, 6-phosphogluconate; 2DDG6P, 2-dehydro-3-deoxy-D-gluconate 6-phosphate; G3P, glyceraldehyde 3-phosphate; PYR, pyruvate; DXP, deoxyxylulose 5-phosphate; MEP, methylerythritol 4-phosphate; CDP-ME, diphosphocytidylyl methylerythritol; CDP-MEP, CDP-ME 2-phosphate; MEC, methylerythritol 2,4-cyclodiphosphate; HMBPP, hydroxymethylbutenyl diphosphate; IPP, isopentenyl diphosphate; DMAPP, dimethylallyl diphosphate; FPP, farnesyl pyrophosphate; OAA, oxaloacetate; CIT, citrate; DTICIT, D-threo-isocitrate; AKG, α-ketoglutarate; SUCCOA, succinyl-CoA; SUCC, succinate; FUM, fumarate; MAL, malate; *dxs*, DXP synthase; *dxr*, DXP reductoisomerase; *ispD*, CDP-ME synthase; *ispE*, CDP-ME kinase; *ispF*, MEC synthase; *ispG*, HMBPP synthase; *ispH*, HMBPP reductase; *ispA*, FPP synthase; *zssI,* α-humulene synthase; *AgBs*, *Abies grandis* α-bisabolene synthase; *ribBG108S*, nDXP enzyme catalyzing the reaction from ribulose 5-phosphate to DXP. Red arrows indicate the overexpression targets.

In our previous study, metabolic flux analysis using a genome-scale model of *M. alcaliphilum* 20Z refined core metabolic pathways of *M. alcaliphilum* 20Z grown on C1 substrates and indicated that a large portion of carbon flux (~75%) enters the RuMP cycle for the regeneration of ribulose-5-phosphate (Ru5P) [13]. Consistent with the highest fluxes of the RuMP cycle, omics datasets also showed higher transcripts, protein abundances, and pool size of metabolites in the RuMP cycle compared to other pathways [12,13]. Therefore, *M. alcaliphilum* 20Z could be a suitable host for producing C5 sugar phosphate-derived products [27]. Based on the in silico analysis of the MTMY_IPP_ and the thermodynamic properties, the MEP pathway is a suitable pathway for isoprenoid production in *M. alcaliphilum* 20Z [19]. However, as noted before, the MEP pathway starts with the condensation of G3P and PYR to produce DXP by DXP synthase (Dxs) which is known as an important control point for the MEP pathway. By using the nDXP route, DXP could be provided directly from C5 sugars. With the high carbon flux through the RuMP cycle, redirection of the carbon flux from the RuMP cycle to the DXP pathway is a promising strategy for improving the carbon flux through the MEP pathway and further enhancing the isoprenoid production (Figure 1). In order to confirm this hypothesis, we performed the flux balance analysis to calculate the MTMY_IPP_ in the presence of the nDXP pathway. The nDXP reaction was manually added to the *i*IA407 model and the first enzyme (Dxs) that condenses G3P and PYR in the MEP pathway was inactivated before simulations (Appendix A). As a result, *M. alcaliphilum* 20Z employing the nDXP pathway resulted in the maximum theoretical molar IPP yield of 14.4% per mmol methane, which was slightly higher than that of the MEP pathway which was 13.6%, revealing that the nDXP route is a more efficient pathway for isoprenoid production in *M. alcaliphilum* 20Z as well as in other type I methanotrophs. Therefore, the synergy between the MEP and nDXP pathways is potentially more appealing to engineering isoprenoids in *M. alcaliphilum* 20Z.

**Figure 2:**
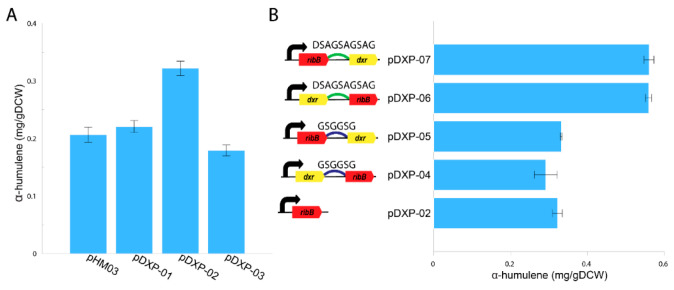
Production of α-humulene in the *M. alcaliphilum* 20Z pHM03, pDXP-01, pDXP-02, and pDXP-03 strains using methane (50% *v*/*v*) as the sole carbon substrate (**A**). Schematic overview of the protein fusions between *ribBG108S* and *dxr* and the α-humulene production of the *M. alcaliphilum* 20Z pDXP-02, pDXP-04, pDXP-05, pDXP-06 and pDXP-07 strains (**B**). Analysis of α-humulene in the dodecane layer was performed, and the concentrations were compared after 96-h cultivation. The data represent the means from three replicates ± standard deviations.

Recently, a novel route for synthesizing DXP from ribulose 5-phosphate was discovered using the directed evolution approach which uncovered two nDXP genes: *yajO* encoding putative xylose reductase and mutant of *ribBG108S* encoding 3,4-dihydroxy-2-butanone 4-phosphate synthase which involved riboflavin biosynthesis [21]. Therefore, to demonstrate the potential use of the nDXP pathway to enhance the production of isoprenoids, we chose to overexpress these nDXP enzymes in *M. alcaliphilum* 20Z (Figure 1). Besides, we selected α-humulene as the target sesquiterpenoid product. In our previous study, for the production of α-humulene, we constructed a pHM03 vector containing α-humulene synthase (*zssI*) along with some bottlenecks of the MEP pathway in *M. alcaliphilum* 20Z (Dxs, IspG, and IspA) that had previously shown to improve the flux pathway [19]. Then, *yajO* and the *ribBG108S* variant from *E. coli* which was generated by Gibson assembly-based site-directed mutagenesis were cloned into pHM03 under control of a strong constitutive P_tac_ promoter, resulting in the pDXP-01 and pDXP-02 vectors (Supplementary Tables S1 and S2). Additionally, we also found other nDXP candidates for screening. The RibB encoding 3,4-dihydroxy-2-butanone 4-phosphate synthase in the archaebacterium *Methanococcus jannaschii* was well-investigated with the availability of the crystal structure [28]. Multiple alignment was performed using RibB from *E. coli* and other well-studied RibB including RibB from *M. jannaschii*, which showed that the amino acid G at position 108 in the RibB from *E. coli* is conserved among these species (Supplementary Figure S1). Therefore, we hypothesized that the replacement of amino acid G to S in other RibB variants might work as nDXP enzymes. Thus, we employed the *ribBG113S* variant from *M. jannaschii* as an nDXP enzyme, resulting in vector pDXP-03 (Appendix B). The resultant strain (pDXP-01-03) was employed using a two-phase culture wherein 20% dodecane was used as the organic phase and methane was used as the carbon source, and α-humulene production in these engineered strains was assayed. Interestingly, as expected, the overexpression of *ribBG108S* and *yajO* from *E. coli* boosted higher α-humulene production compared to the parent strain pHM03, while *ribBG113S* from *M. jannaschii* did not (Figure 2A). In particular, pDXP-02 harboring *ribBG108S* from *E. coli* showed the highest improvement, which was ~1.6-fold higher than the pHM03 strain with the productivity of 0.32 mg/gDCW. In agreement with this result, the previous study had shown that *ribBG108S* has a more efficient enzyme activity for the conversion of Ru5P to DXP as compared to *yajO*. Therefore, the pDXP-02 strain was used for further experiments.

### 3.2. Fusion of nDXP/Dxr Enhanced the Carbon Flux through the nDXP Pathway

There are several protein engineering strategies employed to improve isoprenoid production [29]. Protein fusions or scaffolds were reported to enhance the carbon flux in several cases such as isoprenoid pathway engineering [30]. Due to poor kinetics of RibBG108S enzymes, we hypothesized that protein fusion of RibBG108S and DXP reductase (Dxr) might improve the kinetic property in the delivery of Ru5P to the MEP pathway [21]. Therefore, the fusions of *ribBG108S* and native *dxr* were constructed with different orders and linkers. We used two peptide linkers: G2 corresponds to a GSGGSG linker and DSAG corresponds to a DSAGSAGSAG linker. As a result, the four fused proteins were constructed including *dxr*-G2-*ribB*, *ribB*-G2-*dxr*, *dxr*-DSAG-*ribB*, and *ribB*-DSAG-*dxr* (Figure 2B). These fused proteins were co-overexpressed with the pHM03 vector, resulting in pDXP04–pDXP07 vectors (Supplementary Tables S1 and S2). These vectors also harbored a strong constitutive P_tac_ promoter, which was applied to heterologous expression in *M. alcaliphilum* 20Z. Subsequently, the production of α-humulene from methane in these engineering strains was assayed using aqueous–organic two-phase flask cultivation where 20% dodecane was added as the organic phase. As shown in Figure 2B, the fusions of *ribBG108S* and *dxr* using the DSAG linker (*ribB*-DSAG-*dxr*, *dxr*-DSAG-*ribB*) both yielded higher productivity, which was ~2.8-fold higher than that of the pHM03 strain with the productivity of ~0.56 mg/gDCW. In contrast, the fusions by the G2 linker did not improve the production of α-humulene, suggesting that the G2 linker might not work in *M. alcaliphilum* 20Z. Likewise, Kirby et al. also reported that the expression of the Dxr-RibB (G108S) fusion improved α-bisabolene titers more than fourfold [21]. In summary, these results suggest that the fusion of *dxr* to *ribBG108S* could enhance the carbon flux through the isoprenoid biosynthesis pathway in *M. alcaliphilum* 20Z.

### 3.3. Engineering of M. alcaliphilum 20Z for α-Bisabolene Production via Synergy of the MEP and nDXP Pathways

To expand the suite of products that can be generated from methane using methanotrophic biocatalysts, we further demonstrated the bioconversion of methane to another sesquiterpenoid, α-bisabolene. It is a monocyclic sesquiterpene and a candidate biodiesel fuel that is produced by several microbial hosts via the heterologous expression of α-bisabolene synthase from various plant sources [31]. Recently, to improve the economic feasibility of the biological production of α-bisabolene, various microbial hosts using different alternative carbon sources were used to produce α-bisabolene via the MEP pathway [24,32,33]. However, α-bisabolene production from the next-generation feedstock, such as C1 compounds, is still in its infancy.

**Figure 3:**
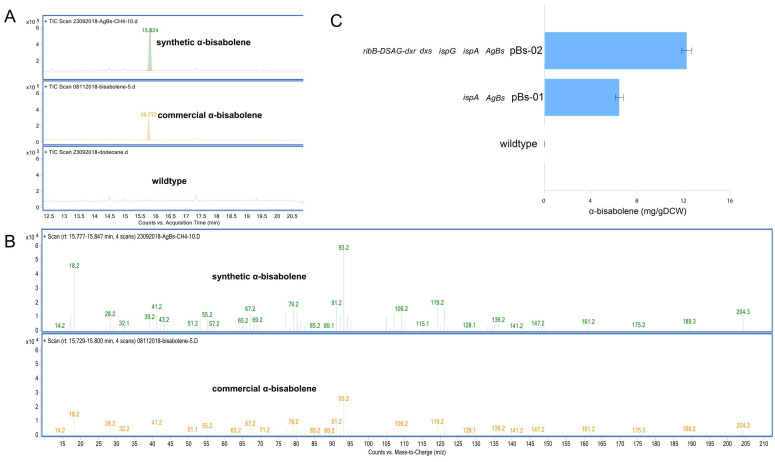
GC-MS chromatogram (**A**), extracted ion GC-MS chromatograms (**B**) of the α-bisabolene standard and synthetic α-bisabolene produced by the pBs-01 strain and α-bisabolene production of the *M. alcaliphilum* 20Z pBs-01 and pBs-02 strains using methane (50% *v*/*v*) as the sole carbon substrate (**C**). Analysis of α-bisabolene in the dodecane layer was performed, and the concentrations were compared after 96-h cultivation. The data represent the means from three replicates ± standard deviations.

To further increase the supply of the farnesyl pyrophosphate (FPP) pool, the native FPP synthase (*ispA*) from *M. alcaliphilum* 20Z was expressed along with α-bisabolene synthase from *Abies grandis* (*AgBs*). The pBs01 vector was constructed for expression of a codon-optimized *AgBs* and native *ispA*, driven by the P_tac_ promoter (Supplementary Tables S1 and S2). Interestingly, the pBs-01 strain could produce α-bisabolene as characterized by GC-MS analysis. As shown in Figure 3A, B, α-bisabolene produced from pBs-01 has a similar retention time and mass spectrum compared to the α-bisabolene standard. In contrast, α-bisabolene was not produced in wildtype. In particular, the pBs-01 strain was able to produce 6.44 ± 0.33 mg/gDCW (12.8 ± 0.66 mg/L) of α-bisabolene (Figure 3C).

As noted in the previous section, the utility of nDXP along with the MEP pathway could improve isoprenoid production using a methanotrophic biocatalyst. Therefore, we expected that the synergy of the DXP route along with the optimized MEP pathway might also enhance α-bisabolene production in *M. alcaliphilum* 20Z. Thus, we constructed a vector for the expression of *AgBs* along with MEP pathway enzymes (*ispA*, *dxs*, and *ispG*) and the optimized nDXP route (*dxr*-DSAG-*ribB*), resulting in the pBs-02 vector (Supplementary Tables S1 and S2). As a result, α-bisabolene was produced in the pBs-02 strain with the titer of 12.24 ± 0.43 mg/gDCW (24.55 ± 0.86 mg/L), which was twofold higher than that in the pBs-01 strain (Figure 3C). These results suggested that the engineering strategies proposed in this study could become a powerful approach for enhancing the production of isoprenoid-related products.

However, in comparison with other conventional bacteria, the resulting titer of isoprenoids from methanotrophic bacteria was still far below. Methanotrophic bacteria utilize methane as the sole carbon source through the oxidation process, which requires a large amount of energy [34]. Besides, the generation of multi-carbon products such as isoprenoids from a C1 compound such as methane or methanol is very difficult. Therefore, the heterologous expression of the assimilation pathway for utilizing an additional carbon source such as xylose beside methane and the addition of a micromineral such as tungsten into the media could be fantastic options to significantly enhance the production of isoprenoids in methanotrophic bacteria. Indeed, the addition of xylose, as well as of tungsten, led to the increase in the growth of methanotrophic bacteria resulting in the improvement of some valuable products such as shinorine, acetoin, 2,3-butanediol, or 3-hydroxybutyric acid [27]. Moreover, for further improvement of the isoprenoid titer, a fed-batch bioreactor with a continuous supply of methane could also be applied [35].

## 4. Conclusions

In summary, we demonstrated a novel engineering strategy for enhancing sesquiterpenoid production from methane in metabolically engineered methanotrophic bacteria by the synergy of the MEP pathway and a novel short-cut nDXP pathway. This study also expanded the suite of sesquiterpenoids converted from methane using methanotrophic biocatalysts. The approaches developed in this study enabled enhanced sesquiterpenoid production and can be applied to the production of diverse isoprenoids from methane.

## Data Availability

The data presented in this study are included in the manuscript, supplementary data, Appendix A and Appendix B.

## Supplementary Materials

The following are available online at https://www.mdpi.com/article/10.3390/microorganisms9061236/s1, Figure S1: Multiple alignment of some RibB variants, Table S1: Strains and plasmids used in this study, Table S2: Primers and procedures used for plasmid construction in this study.

## Appendix A

Genome-scale model of iIA407-nDXP.

## Appendix B

Codon-optimized ribB from *Methanococcus jannaschii*.
