# Supplementary material for: Enhancing Sesquiterpenoid Production from Methane via Synergy of the Methylerythritol Phosphate Pathway and a Short-Cut Route to 1-Deoxy-D-xylulose 5-Phosphate in Methanotrophic Bacteria
Source: Microorganisms. 2021 Jun 7;9(6):1236. doi: 10.3390/microorganisms9061236 (PMC8227265; doi:10.3390/microorganisms9061236)
Supplement: Supplementary file 1 [file microorganisms-09-01236-s001.zip › microorganisms-1234780-supplementary.pdf]

**Supplementary Material**

# Enhancing Sesquiterpenoid Production from Methane via Synergy of the Methylerythritol Phosphate Pathway and a Short-Cut Route to 1-Deoxy-D-xylulose 5-Phosphate in Methanotrophic Bacteria

Anh Duc Nguyen, Diep Ngoc Pham, Tin Hoang Trung Chau, Eun Yeol Lee*

*Department of Chemical Engineering, Kyung Hee University (Integrated Engineering), Yongin-si, Gyeonggi-do 17104, South Korea*

***Corresponding author:** eunylee@khu.ac.kr;

Tel: +82-31-201-3839; Fax: +82-31-204-8114

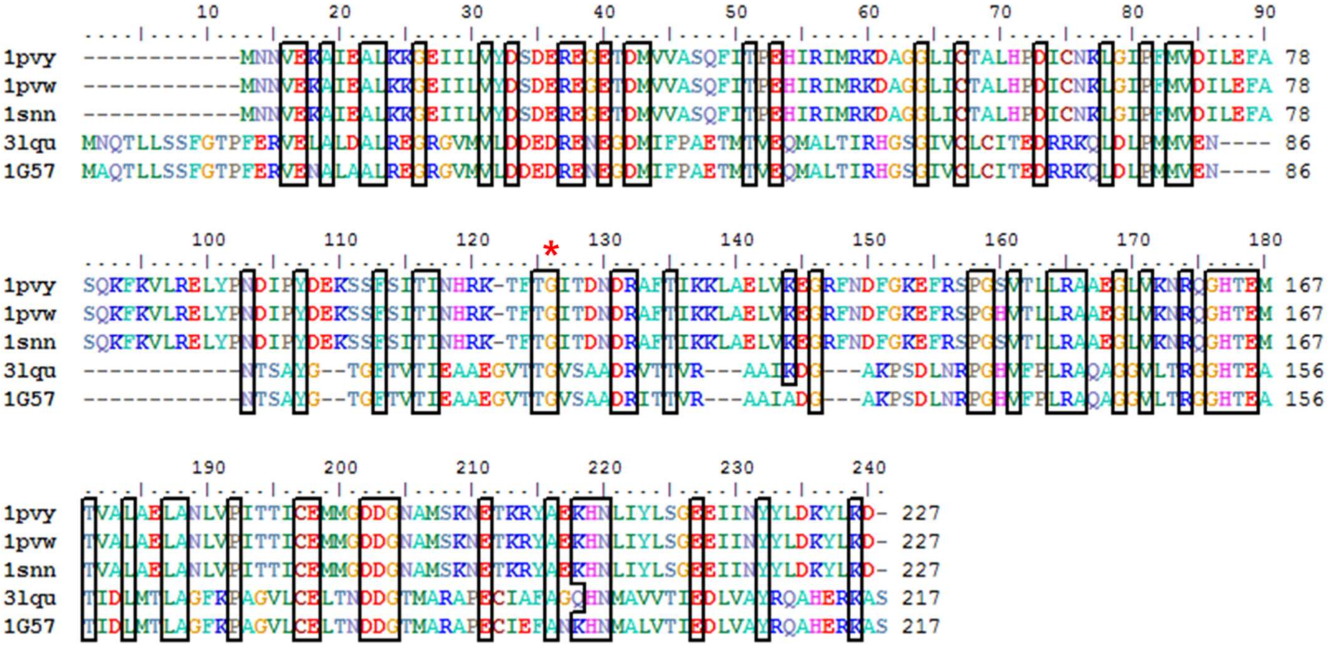


1G57 : RibB of *E. coli* strain K12
1PVW : RibB of *Methanococcus jannaschii*
1PVY, 1SNN : RibB S147H *mutant of Methanococcus jannaschii*
3LQU : RibB of *Salmonella typhimurium*

**Figure S1.** Multiple alignment of some *ribB* variants from different species.

**Table S1**. Strains and plasmids used in this study

| Strain | Characteristic(s) | Reference |
|---|---|---|
| Plasmids | | |
| pHM03 | P*tac*:: *zssI*(opt)-*ispA*(20Z)-*dxs*(20Z)-*ispG*(20Z); oriV oriT trfA; KanR | [1] |
| pDXP-01 | P*tac*:: *zssI*(opt)-*ispA*(20Z)-*dxs*(20Z)-*ispG*(20Z)-*yajO*(*E. coli*); oriV oriT trfA; KanR | This work |
| pDXP-02 | P*tac*:: *zssI*(opt)-*ispA*(20Z)-*dxs*(20Z)-*ispG*(20Z)- *ribBG108S*(*E. coli*); oriV oriT trfA; KanR | This work |
| pDXP-03 | P*tac*:: *zssI*(opt)-*ispA*(20Z)-*dxs*(20Z)-*ispG*(20Z)-*ribBG108S*(*Methanococcus jannaschii*); oriV oriT trfA; KanR | This work |
| pDXP-04 | P*tac*:: *zssI*(opt)-*ispA*(20Z)-*dxs*(20Z)-*ispG*(20Z)- fused *dxr-G2-ribB*; oriV oriT trfA; KanR | This work |
| pDXP-05 | P*tac*:: *zssI*(opt)-*ispA*(20Z)-*dxs*(20Z)-*ispG*(20Z)- fused *ribB-G2-dxr*; oriV oriT trfA; KanR | This work |
| pDXP-06 | P*tac*:: *zssI*(opt)-*ispA*(20Z)-*dxs*(20Z)-*ispG*(20Z)- fused *dxr-DSAG-ribB*; oriV oriT trfA; KanR | This work |
| pDXP-07 | P*tac*:: *zssI*(opt)-*ispA*(20Z)-*dxs*(20Z)-*ispG*(20Z)- fused *ribB-DSAG-dxr*; oriV oriT trfA; KanR | This work |
| pTrcAgBIS (CO) | pTrc99a backbone, $Amp^R$, containing bisabolene synthase from *Abies grandis* | [2] |
| pBs-01 | P*tac*:: *AgBs*(*Abies grandis*)-*ispA*(20Z); oriV oriT trfA; KanR | This work |
| pBs-02 | P*tac*:: *AgBs*(*Abies grandis*)-*ispA*(20Z)-*dxs*(20Z)-*ispG*(20Z)- fused *ribB-DSAG-dxr*; oriV oriT trfA; KanR | This work |

| Strains | | |
|---|---|---|
| *Escherichia coli* DH5α | | Novagen |
| *Methylotuvimicrobium alcaliphilum* 20Z | Used as the host strain | DMSZ |
| pDXP-02 | *M. alcaliphilum* 20Z harboring pDXP-02 vector | This work |
| pDXP-03 | *M. alcaliphilum* 20Z harboring pDXP-03 vector | This work |
| pDXP-04 | *M. alcaliphilum* 20Z harboring pDXP-04 vector | This work |
| pDXP-05 | *M. alcaliphilum* 20Z harboring pDXP-05 vector | This work |
| pDXP-06 | *M. alcaliphilum* 20Z harboring pDXP-06 vector | This work |
| pDXP-07 | *M. alcaliphilum* 20Z harboring pDXP-07 vector | This work |
| pBs-01 | *M. alcaliphilum* 20Z harboring pBs-01 vector | This work |
| pBs-02 | *M. alcaliphilum* 20Z harboring pBs-02 vector | This work |

**Table S2**. Primers used and procedures for plasmids construction in this study

| Primer | Sequence | Description |
|---|---|---|
| pAWP89-For | TAGTTGTCGGGAAGATGCGT | For amplifying pAWP89 backbone which contained $P_{tac}$ promoter and SD sequence |
| pAWP89-Rev | AGCTGTTTCCTGTGTGAATA | |
| ispG-invert-Rev | CTACTGAGCTGTGGTTCCGT | For linear pHM03 vector |
| pAWP89-For | TAGTTGTCGGGAAGATGCGT | |
| agB.s-For | TTCACACAGGAAACAGCTATGGCGGGTGTTTCTGCG | For construction of pBs-01 vector. AgB.s and ispA from 20Z were amplified and ligated into pAWP89 backbone. |
| agB.s-Rev | ACTCAGATCTTACAGCGGCAGCGGTTC | |
| ispA-20Z-For | CCGCTGTAAGATCTGAGTACCTCTAGAAAATAAGGA | |
| ispA-20Z-Rev | GCATCTTCCCGACAACTATTAATGATCTCGCTGGATAA | |
| ribB-KpnI-For | CTGGTCTGTTTGTGGTACAGGAAACAGCTATGAATCAGACGCTACTTTCCTCT | For construction of ribB-G108S from *via* site-directed mutagenesis. RibB-G108S and yajO were first cloned into pLC291 vector at KpnI and SacI restriction sites. |
| ribB-KpnI-Rev | CGTAGATCTTCTAGAGGTACTCAGCTGGCTTTACGCTCAT | |
| ribB-G108S-Rev | GGCAGAAACCGAGGTAGTCA | |
| ribB-G108S-For | TGACTACCTCGGTTTCTGCC | |
| yajO-SacI-For | TACTAGTGCATGCGAGCTAAGGAGATATAATGCAATACAACCCCTTAGGA | |
| yajO-SacI-Rev | TGAATTCACCGGTGAGCTTTATTTAAATCCTACGACAGGATGCG | |
| ribB-89-For | CGGAACCACAGCCAGTAGAGGAAACAGCTATGAATCAGACGCTACTTTC | For construction of pDXP-06 and pDXP-07 vectors |
| ribB-DSAG-Rev | ACCAGCACTACCAGCACTACCAGCACTATCGCTGGCTTTACGCTCATGTG | |
| ispC-DSGA-For | GATAGTGCTGGTAGTGCTGGTAGTGCTGGTAAAGGTATTTGTATTTTGGGCGC | |
| ispC-89-Rev | GCATCTTCCCGACAACTATTAACGCTTAAGTTCTTCGACGA | |
| ispC-DSAG-ribB-Rev | ACCAGCACTACCAGCACTACCAGCACTATCACGCTTAAGTTCTTCGACGA | |
| ispC-DSAG-ribB-For | GATAGTGCTGGTAGTGCTGGTAGTGCTGGTAATCAGACGCTACTTTCCTCT | |
| ispC-89-For | CGGAACCACAGCTCAGTAGAGGAAACAGCTATGAAAGGT | |

| | | |
|---|---|---|
| ribB-89-Rev | GCATCTTCCCGACAACTATCAGCTGGCTTTACGCTCAT | |
| yajO-89-For | CGGAACCACAGCTCAGTAGAAGGAGATATAATGCAATACAACCCC | For construction of pDXP-02 vector |
| yajO-89-Rev | GCATCTTCCCGACAACTATTATTTAAATCCTACGACAGGATGCG | |
| ribB-89-Rev | GCATCTTCCCGACAACTATCAGCTGGCTTTACGCTCAT | For construction of pDXP-01 vector |
| ribB-89-For | CGGAACCACAGCCAGTAGAGGAAACAGCTATGAATCAGACGCTACTTTC | |
| ribB-89-For | CGGAACCACAGCCAGTAGAGGAAACAGCTATGAATCAGACGCTACTTTC | For construction of pDXP-04 and pDXP-05 vectors |
| ribBG2ispC-Rev (G2 fused) | ACCGGAACCGCCAGAACCGCTGGCTTTACGCTCATGTG | |
| ribBG2ispC-For (G2 fused) | GGTTCTGGCGGTTCCGGTAAAGGTATTTGTATTTTGGGCGC | |
| ispC-89-Rev | GCATCTTCCCGACAACTATTAACGCTTAAGTTCTTCGACGA | |
| ispC-89-For | CGGAACCACAGCTCAGTAGAGGAAACAGCTATGAAAGGT | |
| ribB-89-Rev | GCATCTTCCCGACAACTATCAGCTGGCTTTACGCTCAT | |
| CO_ribB_G113S_For | CGGAACCACAGCTCAGTAGAATAAGGAGGAATAAACCATGAATA ATGTCGAAAAGGCGATCG | For construction of pDXP-03 vector |
| CO_ribB_G113S_Rev | GCATCTTCCCGACAACTAGTCTTTCAGATATTTATCCAAA | |

**APPENDIX B**. Codon-optimized *ribB* from *Methanococcus jannaschii*

>*ribBG113S*

ATGAATAATGTCGAAAAGGCGATCGAAGCGTTGAAAAAAGGCGAGATCATTTTGGTTTACGATAGCGATG
AACGCGAGGGCGAAACCGATATGGTGGTCGCGTCGCAATTTATCACGCCGGAACATATCCGCATCATGCG
CAAAGATGCCGGCGGCTTGATTTGTACCGCATTGCATCCGGACATCTGCAATAAATTGGGCATTCCGTTCA
TGGTCGATATCTTGGAATTTGCCAGCCAAAAATTCAAAGTCTTGCGCGAACTGTATCCGAACGATATTCCT
TATGACGAAAAATCGTCGTTTTCGATTACCATTAATCATCGAAAAACCTTTACCTCGATCACCGACAACGA
CCGCGCGTTTACGATCAAAAAGCTGGCCGAGCTGGTCAAGGAAGGCCGCTTCAATGATTTCGGCAAAGA
ATTCCGCTCGCCGGGTCATGTCACCTTGCTGCGCGCCGCCGAAGGTTTGGTCAAAAATCGGCAAGGCCAT
ACCGAAATGACCGTCGCCTTGGCGGAATTGGCCAATTTGGTGCCGATTACGACGATTTGCGAAATGATGG
GCGATGACGGCAATGCGATGTCGAAAAACGAAACGAAACGCTATGCCGAAAAACATAATCTGATTTATT
TAAGCGGCGAAGAAATCATCAATTATTATTTGGATAAATATCTGAAAGAC

## References

[1] Nguyen AD, Kim D, Lee EY. Unlocking the biosynthesis of sesquiterpenoids from methane via the methylerythritol phosphate pathway in methanotrophic bacteria, using α-humulene as a model compound. Metab Eng 2020;61:69-78.
[2] Peralta-Yahya PP, Ouellet M, Chan R, Mukhopadhyay A, Keasling JD, Lee TS. Identification and microbial production of a terpene-based advanced biofuel. Nat Commun 2011;2:1-8. 10.1038/ncomms1494
